# Hand weakness in Charcot-Marie-Tooth disease 1X

**DOI:** 10.1016/j.nmd.2012.02.008

**Published:** 2012-07

**Authors:** P.J. Arthur-Farraj, S.M. Murphy, M. Laura, M.P. Lunn, H. Manji, J. Blake, G. Ramdharry, Z. Fox, M.M. Reilly

**Affiliations:** MRC Centre for Neuromuscular Diseases, Department of Molecular Neuroscience, UCL Institute of Neurology and the National Hospital for Neurology and Neurosurgery, Queen Square, London, UK

**Keywords:** Charcot–Marie–Tooth disease, Connexin 32, Hand strength, Overwork weakness, Neuropathy, GJB1

## Abstract

There have been suggestions from previous studies that patients with Charcot–Marie–Tooth disease (CMT) have weaker dominant hand muscles. Since all studies to date have included a heterogeneous group of CMT patients we decided to analyse hand strength in 43 patients with CMT1X. We recorded handedness and the MRC scores for the first dorsal interosseous and abductor pollicis brevis muscles, median and ulnar nerve compound motor action potentials and conduction velocities in dominant and non-dominant hands. Twenty-two CMT1X patients (51%) had a weaker dominant hand; none had a stronger dominant hand. Mean MRC scores were significantly higher for first dorsal interosseous and abductor pollicis brevis in non-dominant hands compared to dominant hands. Median nerve compound motor action potentials were significantly reduced in dominant compared to non-dominant hands. We conclude that the dominant hand is weaker than the non-dominant hand in patients with CMT1X.

## 1 Introduction

The degree of hand weakness in neurological diseases such as Charcot–Marie–Tooth disease (CMT) is of particular importance since it has a large impact on day to day functioning and quality of life [1]. A study of 124 patients suggested that patients with Charcot–Marie–Tooth disease (CMT) might be susceptible to overwork weakness when comparing the strength of dominant to non-dominant hand muscles [2]. However, a further study using hand dynamometry on 28 patients with CMT did not show any significant difference in muscle strength between dominant and non-dominant hands [3].

Overwork weakness in neuromuscular disease remains a contentious issue. Initially it was used to explain apparent activity-dependent weakness that developed in patients with post-polio syndrome [4]. It has also been described in patients with facioscapulohumeral muscular dystrophy [5]. However, trials of exercise in patients with CMT, myotonic dystrophy, Guillain–Barré syndrome, chronic inflammatory polyradiculoneuropathy (CIDP) and even post-polio syndrome have not shown deterioration in muscle strength [6–8].

Charcot–Marie–Tooth (CMT) disease is a genetically heterogeneous inherited neuropathy. Over 300 mutations have been described in the *Gap Junction Beta 1* (*GJB1*) gene that cause an X-linked form of the disease (CMT1X), the second commonest form of CMT [9] (http://www.molgen.ua.ac.be/CMTMutations). Mutations in *GJB1* cause neuropathy through a loss of normal gene function [10]. *GJB1* codes for a protein called Connexin 32. It is expressed by both Schwann cells and oligodendrocytes and is thought to act as a gap junction protein, though how loss of normal function leads to axon loss is yet to be elucidated [9]*.*

Clinically, patients with CMT1X have a progressive motor and sensory neuropathy with distal wasting. Some patients also develop a central nervous system phenotype, which can either present acutely or chronically [11]. Males are typically more severely affected than females [12]. Electrophysiological studies demonstrate intermediate slowing of conduction velocities with features of demyelination and axonal loss and a variation in involvement within and between nerves [13].

Since previous studies investigating hand strength in CMT have included a genetically mixed group of CMT patients, we decided to look at hand muscle strength in a single group of CMT patients, CMT1X. The aim of this study was to determine whether there was any difference in the strength of the muscles in patients with CMT1X between dominant and non-dominant hands. We also aimed to determine whether there were differences in strength between hand muscles innervated by different nerves as has been described previously with abductor pollicis brevis (APB) being weaker than first dorsal interosseous (FDIO) [9,14]. For both analyses we examined whether nerve conduction studies correlated with our muscle strength findings.

## 2 Methods

We retrospectively reviewed the notes of 53 patients attending the National Hospital for Neurology and Neurosurgery with a genetically confirmed diagnosis of CMT1X.

We recorded handedness, age, gender, mutation, most recent CMT neuropathy score (CMTNS) [15], most recent MRC score for FDIO and APB and most recent median and ulnar nerve compound motor action potentials (CMAPs) and conduction velocities. Median CMAP was recorded from the APB and ulnar CMAP from the abductor digiti minimi (ADM). For numerical analysis, MRC strength scores of 4− and 4+ were converted to values of 3.5 and 4.5, respectively.

The FDIO score for the dominant hand was tabulated against the FDIO score for the non-dominant hand and distributions were examined. In order to examine whether there was a difference in FDIO strength between hands, various MRC scores were tested as a cut off. For example, an FDIO score cut-off of 4− meant, we categorised scores into ⩽4− versus >4−. These categories were compared between the dominant and non-dominant hands using a McNemar’s test to see whether the same proportion of scores were considered to be ⩽4− for both hands. Similar comparisons were made for APB MRC scores.

In order to make comparisons between the dominant and non-dominant hands in nerve conduction outcomes and in MRC strength scores, the distribution of the difference in outcome values between hands was assessed. Non-parametric Wilcoxon sign-rank tests (i.e. matched-pairs tests) were used to compare skewed differences and paired *t*-tests were used for normally distributed differences. Multilevel models were performed to account for the non-independence of observations in the dominant and non-dominant hands, where clustering was assumed to have occurred within each patient [16]. These multilevel models explore the relationship between each of the outcomes with hand-dominance after adjustment for age and gender. A *p* value <0.01 was considered significant.

All statistical analyses were performed using Stata (StataCorp. 2009. *Stata Statistical Software: Release 11*. College Station, TX: StataCorp LP).

## 3 Results

Of the 53 patients, nine were excluded because MRC score was not adequately documented and a further one was discounted due to having an additional diagnosis of CIDP. This left 43 patients, 22 males and 21 females, with 29 different mutations in the *GJB1* gene (Table 1). Males were statistically younger, with a mean age of 37.2 years compared to 45.4 years for females (*p* < 0.01). Males had a mean CMTNS of 15.2 (*n* = 10, SD +/−4) and females a CMTNS of 12.6 (*n* = 13, SD +/−4.4). Seventy-three percent of patients were examined by a single author (MMR).

In CMT1X patients, FDIO strength was higher in the non-dominant hand for 15/43 (34.9%) individuals and APB strength was higher in the non-dominant hand for 14/43 (32.6%) individuals. There were 22/43 (51.2%) patients with a weaker dominant hand based on a combined MRC score of FDIO and APB. No patient was found to have a stronger dominant hand for either FDIO or APB MRC strength scores individually or for the combined score.

When we categorised individuals into MRC strength scores of >4− and ⩽4−, there was a significant difference in strength according to hand-dominance. We identified nine (20.9%) individuals who had a non-dominant FDIO score >4− and a dominant FDIO score ⩽4− (*p* = 0.003) and eight (18.6%) patients who had a non-dominant APB score >4− and a dominant APB score ⩽4− (*p* = 0.005). Using other MRC grades as a cut off did not show statistical significance in our sample population.

The mean strength scores for FDIO and APB were significantly higher in non-dominant compared to dominant hands (*p* < 0.0001; Table 2). After adjustment for age and gender there was a mean 0.22 (95% CI: 0.12, 0.32) higher FDIO score for non-dominant hands compared to dominant hands and in a separate model there was a 0.30 (95% CI: 0.14, 0.47) higher APB score for non-dominant hands compared to dominant hands. In addition, males had a −1.14 (95% CI: −1.77, −0.52) lower APB score than females.

Median nerve CMAPs were smaller in dominant hands compared to non-dominant hands, though this difference did not achieve statistical significance, most likely due to the small sample size (*n* = 18; *p* = 0.02; Table 2). There was no significant difference in ulnar nerve CMAPs between dominant and non-dominant hands (*n* = 14; *p* = 0.89; Table 2). There was no difference between dominant and non-dominant hands for median and ulnar nerve conduction velocities (data not shown).

When comparing FDIO to APB strength in the dominant hand of CMT1X patients, we found that APB was significantly weaker (*p* = 0.001; Table 3A). After adjustment for age and gender, there was a mean −0.40 difference in score for APB compared to FDIO (95% CI: −0.64, −0.15; *p* = 0.001). Furthermore, the associated nerve conduction studies demonstrated that median nerve CMAPs (APB) were significantly lower than the ulnar nerve CMAPs (ADM) in CMT1X patients (*p* < 0.0001; Table 3B). Similarly, conduction velocities were significantly slower in median nerves compared to ulnar nerves (*p* = 0.0005; Table 3B).

## 4 Discussion

We reviewed the notes of 43 patients with CMT1X and demonstrated that patients with CMT1X had a weaker dominant hand. Median nerve CMAPs were reduced in the dominant compared to the non-dominant hand. Furthermore, we showed that the APB muscle was weaker than FDIO, confirming previous suggestions that there is a greater impact on median nerve function compared to the ulnar nerve [9,14].

There are several limitations to note with this study. Firstly, we used the MRC scoring system to measure hand strength. A recent study demonstrated that while there is a significant correlation between MRC scoring for hand muscles and muscle strength measured by the Rotterdam intrinsic hand myometer the scale is not linear and there is considerable overlap between MRC grades 4 and 5 [17]. However, the cut off for significance occurred around MRC grade 4− in our data set suggesting that the boundary between grades 4 and 5 was not crucial for demonstrating difference in strength between dominant and non-dominant hands. One would expect that overwork would exert its effects over time so that differences should increase with age; however, our patient sample size was not large enough to assess the effect of patient age, or disease severity, measured by the CMTNS, on hand strength. Additionally, a larger patient sample would be necessary to analyse the effect of gender on hand strength although our data was suggestive that the difference between dominant and non-dominant hands was present in males and females for both FDIO and APB muscles (data not shown). We demonstrated a reduction in median nerve CMAP in the dominant hand; however, only 18 of 43 patients had both right and left median nerves assessed, thus this result was not statistically significant. We did not show any difference between dominant and non-dominant ulnar nerve CMAPs but this may be due to the ulnar nerve being less affected in CMT1X [12]. Conduction velocity measurements showed no difference between dominant and non-dominant hands. This was unsurprising since CMAP values and not conduction velocities have been shown to correlate with muscle weakness and disease progression [10,18]. In addition, although we used FDIO MRC score for the strength scoring, the ADM was used to determine ulnar CMAP.

Reassuringly, our study has confirmed several previous observations reported in CMT1X patients. Firstly we have shown that APB is weaker in males than in females. Additionally, our male subgroup was significantly younger and had a higher mean CMTNS. These findings all fit with males being more severely affected with an earlier disease onset [10,12]. We also showed that median nerve CMAPs and conduction velocities were reduced compared to the ulnar nerve consistent with the clinical observation of APB weakness being greater than the ulnar innervated hand muscles, confirming previous findings [9,12,14].

Although the results in this study suggest the dominant hand is weaker in patients with CMT1X, these results should be confirmed by a prospective blinded study, ideally using muscle myometry, MRC scoring and nerve conduction studies. However, this study does highlight the need to investigate hand strength in an aetiologically homogenous population since previous hand strength studies in CMT have used a mixed population of patients with all forms of CMT [2,3].

It has been suggested in previous studies that a difference in hand strength between dominant and non-dominant hands may suggest that overwork weakness causes increased muscle weakness as the dominant hand will be used more by the patient [2]. Whether this extrapolation is valid remains to be seen but it is an important issue as exercise training to improve strength in CMT is a growing area of research. The few studies, at present, do not show any harmful effects of exercise on CMT and may show some subtle improvements [6,19]. However, it is important to note that strengthening exercises in these studies concentrate on proximal muscles whereas the distal muscles are more affected in CMT [20]. While our study does suggest that the dominant hand is weaker in patients with CMT1X, it would be premature to suggest this is due to overwork weakness. Hand weakness in CMT1X has many unexplained facets requiring further studies including the current finding of increased weakness in the dominant hand and also the greater involvement of the median innervated muscles compared to the ulnar innervated muscles as shown in this and previous studies [9,12,14].

## Figures and Tables

**Table 1:** List of mutations in 43 patients studied with CMT1X.

Number	Mutation
1	c.-373G>A (5′UTR)
2	p.Trp3Stop
3	p.Arg15Gln
4	p.Arg22Gln
5	p.Leu25Phe
6	p.Ala39Val
7	p.Ala40Thr
8	p.Ser42CysfsX45
9	p.Cys60Phe
10	p.Tyr65X
11	p.Gln80Lys
12	p.Val91Met
13	p.Val91Leu
14	p.Val91Gly
15	p.His94Arg
16	p.Arg107Trp
17	p.Arg142Gln
18	p.Arg142Trp
19	p.Phe153Ser
20	p.Tyrl 57Cys
21	p.Phe158Ser
22	p.Gly159Asp
23	p.Tyrl 60Cys
24	p.Arg164Gly
25	p.Leu165Pro
26	p.Cys173Arg
27	p.Val192Phe
28	p.Arg215Trp
29	p.Gly21Asn and
	p.Met162Thr (same allele)

**Table 2:** Comparison of hand muscles’ strength and ulnar and median nerve conduction studies between dominant and non-dominant hands.

	Dominant hand	95% CI	Non-dominant hand	95% CI	*P* value
Mean score FDIO	3.78	3.56, 4.00	4.00	3.80, 4.00	<0.0001
Mean score APB	3.38	3.02, 3.75	3.69	3.35, 4.02	0.0001
CMAP median (APB, mV)	2.03	0.96, 3.10	3.03	2.13, 3.93	0.02
CMAP ulnar (ADM, mV)	4.70	3.15, 6.25	4.86	3.48, 6.24	0.89

Patients with CMT1X have a higher mean MRC strength score for the first dorsal interosseous (FDIO) muscle and the abductor pollicis brevis (APB) muscle in non-dominant hands compared to dominant hands. Median nerve compound motor action potentials (CMAP) (measured on APB) were larger in non-dominant hands whereas ulnar nerve CMAPs (measured on abductor digiti minimi, ADM) were similar between hands. Mean values with 95% confidence interval are shown.

**Table 3A:** Comparison of MRC strength scores for FDIO and APB muscles in the dominant hand.

	FDIO	95% CI	APB	95% CI	*P* value
Mean MRC score	3.78	3.56, 4.00	3.38	3.02, 3.75	0.001

Abductor pollicis brevis (APB) muscle is significantly weaker than the first dorsal interosseous (FDIO) muscle in the dominant hand of patients with CMT1X, measured by mean MRC strength score.

**Table 3B:** Comparison of nerve conduction studies between median and ulnar nerves.

	Ulnar nerve (ADM)	95% CI	Median nerve (APB)	95% CI	*P* value
Mean CMAP (mV)	5.53	4.75, 6.31	2.71	1.90,3.51	<0.0001
Mean CV (m/s)	42.89	38.86, 46.93	34.98	31.47,38.48	0.0005

Median nerve Compound motor action potentials (CMAP) and conduction velocities (CV) (both measured on APB) were statistically lower than ulnar nerve CMAPs and conduction velocities (measured on abductor digiti minimi, ADM). Mean values with 95% confidence interval are shown.
